# Pre-Impact Fall Detection: Optimal Sensor Positioning Based on a Machine Learning Paradigm

**DOI:** 10.1371/journal.pone.0092037

**Published:** 2014-03-21

**Authors:** Dario Martelli, Fiorenzo Artoni, Vito Monaco, Angelo Maria Sabatini, Silvestro Micera

**Affiliations:** 1 The BioRobotics Institute, Scuola Superiore Sant'Anna, Pisa, Italy; 2 Translational Neural Engineering Lab, Center for Neuroprosthetics, Swiss Federal Institute of Technology Lausanne (EPFL), Lausanne, Switzerland; The University of Western Ontario, Canada

## Abstract

The aim of this study was to identify the best subset of body segments that provides for a rapid and reliable detection of the transition from steady walking to a slipping event. Fifteen healthy young subjects managed unexpected perturbations during walking. Whole-body 3D kinematics was recorded and a machine learning algorithm was developed to detect perturbation events. In particular, the linear acceleration of all the body segments was parsed by Independent Component Analysis and a Neural Network was used to classify walking from unexpected perturbations. The Mean Detection Time (*MDT*) was 351±123 ms with an *Accuracy* of 95.4%. The procedure was repeated with data related to different subsets of all body segments whose variability appeared strongly influenced by the perturbation-induced dynamic modifications. Accordingly, feet and hands accounted for most data information and the performance of the algorithm were slightly reduced using their combination. Results support the hypothesis that, in the framework of the proposed approach, the information conveyed by all the body segments is redundant to achieve effective fall detection, and suitable performance can be obtained by simply observing the kinematics of upper and lower distal extremities. Future studies are required to assess the extent to which such results can be reproduced in older adults and in different experimental conditions.

## Introduction

Fall-related accidents are among the most serious problems in elderly people [1], [2], [3], amputees [4] and subjects with neurological disorders [5]. About one out of three adults over 65 years of age falls once a year leading to severe traumatic and psychological consequences [1], [2], [3]. Given the increased life expectancy of the worldwide population, the number of people who are more prone to fall is growing and fall-prevention programs are rapidly becoming a key issue to health-care national systems, not only to reduce costs, but also to benefit society as a whole [3].

In the last years, several research groups have focused their attention on the design of “pre-impact fall detectors”, that is, wearable devices able to reveal unexpected and potentially dangerous postural transitions, which can result in a fall. They have sought to detect an incipient fall early enough to enable suitable strategies aimed at mitigating the effects of the impact with the ground or, possibly, avoiding it [6], [7], [8], [9], [10], [11], [12], [13]. For instance, these devices could be combined with air-bag technology [14], [15] to reduce the risk of hip fractures or with exoskeleton/active prosthesis systems that provide supplementary forces to regain stability [13], [16].

An effective pre-impact detection system is expected to both increase the sense of security and reduce fall-related injuries (occurring in 40–60% of cases [1]), ultimately improving users' quality of life. However, in order to increase the efficacy and, consequently, the usability of these devices, some efforts are still needed to overcome the limits of currently adopted approaches.

From a methodological viewpoint, general kinematic features of falling are usually measured from standing position while subjects fall either passively [6], [7], [8], [9] or as a result of pushing [10], [11], that is, in very unnatural conditions. Conversely, falls generally occur during walking [2] and are characterized by a very complex biomechanics which depends on several factors such as the type of perturbation [17], the intensity [18], the direction [19], and the timing with respect to the gait cycle [17], [20]. Consequently, although promising, many of the results reported in literature may be biased by the unnatural behaviour elicited by these experimental setups.

One of the potential weak points of the pre-impact fall detectors is related to the body sensors positioning. To the best of our knowledge, work reported in literature has not yet clarified whether it is possible to determine the body segments which are the most sensitive to postural transitions resulting in falls. Specifically, current approaches are usually based on a small set of inertial sensors fixed to the pelvis [7], [9], [11], the thorax [6], [7], [8], [12], or the thigh [8], [9] with the overall goal to minimize their total number.

Indeed, Zhao and colleagues [21], after recording data from nine sensors positioned on trunk and legs, observed that the acceleration of the chest provides the best performance while detecting a passively induced fall. Moreover, Aziz and colleagues [22] analyzed the acceleration of several body segments in order to reveal, *a posteriori*, whether the falls were induced by slips, trips or other causes. They reported that three markers positioned on both the ankles and the sternum provide the best classification results. However no study has investigated whole-body kinematics to maximize speed and efficiency of a pre-impact fall detector.

The aim of the present study was to investigate whole-body 3D kinematics while managing unexpected perturbations delivered during steady locomotion. Specifically, our goal was to identify the combination of body segments that provides for a rapid and reliable detection of the transition from steady walking to a slipping event. Our hypothesis was that the linear acceleration of a limited set of body segments could be very sensitive to underlying fall-triggering processes in healthy young adults. If confirmed, this would improve the performance of pre-impact fall detectors based on an optimized positioning of the sensors.

Since a great amount of falls and fall-related injuries results from slips [3], an experimental set up was designed to deliver multidirectional slipping-like perturbations to enrolled subjects while walking in a steady state. The 3D kinematics was recorded and a machine learning algorithm was developed to detect perturbation events. In particular, the linear acceleration of all body segments was analyzed with a robust Independent Component Analysis (*ICA*) (to parse the input data into maximally independent components) and a Neural Network (*NN*) was used to classify walking from unexpected perturbations.

The pattern-recognition performance using all the body segments was finally compared with different reduced subsets (singled out by *ICA*) whose variability appeared strongly influenced by the perturbation-induced dynamic modifications.

## Materials and Methods

### Subjects, experimental setting and protocol

Fifteen healthy adults (10 males and 5 females, 26.1±1.3 years old, 68.8±12.3 kg, 1.78±0.06 m, right dominance of the lower limb) underwent slipping perturbations during paced walking. The experimental set-up and the protocol are the same used in our previous study [23] and briefly reported here.

**Figure 1:**
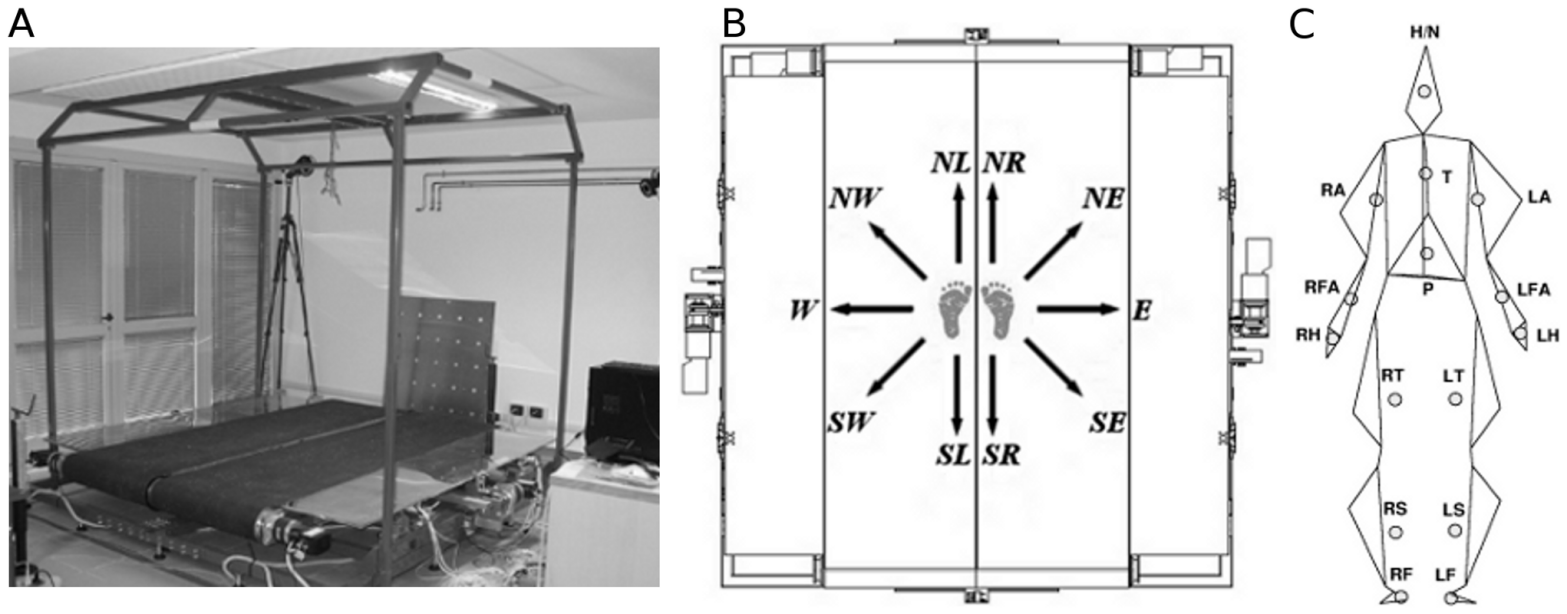
Experimental setup. The subplot A consists of a picture of the SENLY platform. The subplot B represents the 10 types of perturbations. Each perturbation involved the combination of longitudinal (i.e., North, *N* or South, *S*) and transversal (i.e., East, *E*, or West, *W*) movements of the belt provided while participants were walking steadily. Five perturbations were delivered on the left foot (i.e., *NL*, *NW*, *W*, *SW*, *SL*) and five on the right foot (i.e., *NR*, *NE*, *E*, *SE*, *SR*). The subplot C shows an example of the reconstruction of the biomechanical model of a representative subject. The vertexes of each polygon and the dots represent respectively the markers position and the *CoM* of each body segment with the corresponding acronyms.

Perturbations were managed by a mechatronic platform named SENLY [24], which mainly consists of two parallel and adjacent treadmills whose longitudinal and transversal speeds can be independently controlled (Figure 1A). This allows slipping perturbations to be applied to the horizontal plane by means of sudden movements of one or both belts.

The experimental protocol consisted in perturbing the steady locomotion of each subject with a sudden and unexpected movement of one belt occurring at the heel strike. In order to guarantee the same dynamical stability for all the subjects, the walking speed was chosen by imposing a Froude number equal to 0.15 [25]. Each recording session (trial) began while a subject was walking steadily, started one minute before delivering the perturbation, and ended after the recovery of balance. Subjects wore a safety harness attached to an overhead track.

Ten types of perturbations, five on the left (*L*) foot and five on the right (*R*) foot, were delivered. Each involved the combination of longitudinal (North, *N* or South, *S*) and/or transversal (East, *E*, or West, *W*) movements of the belts. Herein, perturbations will be named with these acronyms: *NR*, *NE*, *E*, *SE*, *SR*, *NL*, *NW*, *W*, *SW*, *SL* (Figure 1B).

A 6-camera motion analysis system operating at 100 Hz and a set of 39 markers were used to quantify the whole-body 3D kinematics [23]. The 3D kinematics and the onset of the perturbations were synchronized by means of a logic pulse generated by SENLY. Two sessions were collected for each subject and each perturbation. In order to obtain unbiased results due to habituation, participants did not know whether they would be perturbed or not (ten trials without perturbation were also included in the experimental protocol but not in the data analysis) and perturbations were supplied in random order.

The Local Ethics Committee of the Scuola Superiore Sant'Anna approved the research procedure and all the subjects gave their written informed consent, in agreement with the ethical standards of the declaration of Helsinki, before participating.

### Biomechanical Model

A full body model accounting for 15 segments was developed [23]. The 15 segments (Figure 1C) were: head/neck (*H/N*), chest (*T*), abdomen/pelvis (*P*), upper arms (*LA* and *RA*), forearms (*LFA* and *RFA*), hands (*LH* and *RH*), thighs (*LT* and *RT*), shanks (*LS* and *RS*) and feet (*LF* and *RF*). For each body segment, a right-handed local reference frame was located in its Centre of Mass (*CoM*), calculated using the procedures described by Zatsiorsky and colleagues [26] and modified by de Leva [27]. For each segment, the three components of the linear acceleration of the *CoM* resolved in the global-reference frame were calculated as the second derivative of the position data (three-point central differences method). Only the portion of these signals across the onset of the perturbation was then used in the fall-detection algorithm.

### Data Processing

**Figure 2:**
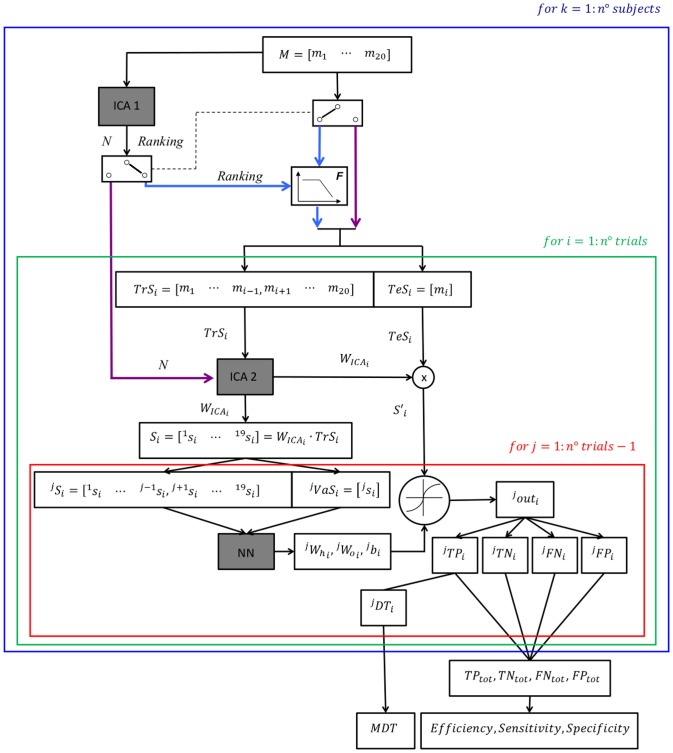
Algorithm. The scheme shows the entire algorithm used in this study. The red, green and blue boxes represent respectively the inner *LOOCV*, the outer *LOOCV* steps and the repetitions for all the subjects. The gray boxes represent the main core of the algorithm (i.e., *ICA1*, *ICA2* and *NN*). The two switches are rigidly interconnected (dotted line) and allow the flow of information coming from the upper layers of the algorithm to go through either the purple lines (i.e., it occurs when the algorithm processes the dataset accounting for all body segments; see the section titled *Processing of the datasets accounting for all segments*) or the blue lines (i.e., it occurs when the algorithm processes the dataset accounting for a subset of all body segments; see the section titled *Processing of the datasets accounting for a subset of all segments*). Note that when the switches are commuted toward the purple (blue) lines no information can go through the blue (purple) lines, that is, the unselected lines are neglected by the algorithm. *M* is the initial dataset constructed by concatenating data related to the 20 collected trials and is preliminarily parsed out by *ICA1* to determine the optimal number *N* of retained *ICs* and the *Ranking* concerning their informativeness. These information were respectively used to run the *ICA2* either on the dataset accounting for all body segments (purple line) or on that accounting for a limited number of segments (light blue line). In this regard the box *F* selects the subset of segments being processed according to their *Ranking*. Then, 19 out of 20 trials (Training Set–*TrS_i_*) are selected to train the model, whereas the excluded trial (Test Set–*TeS_i_*) is used to test its performance. This procedure is repeated 20 times (“outer” *LOOCV*) leaving a different trial as *TeS_i_* each time (i.e., 20 *TrS_i_* and 20 *TeS_i_* for each subject– see green box). In order to train the model, *ICA2* is performed on the *TrS_i_* with the number of components previously determined. *ICA2* produces a time-invariant matrix (*W_ICAi_*) which is used to project both the *TeS_i_* and the *TrS_i_* into the independent component space. The resultant projected *TrS_i_* (*S_i_*) is passed on to the Neural Network (*NN*). A further *LOOCV* step (inner *LOOCV*) is applied to *S_i_* (19 trials): 18 of them (*^j^S_i_*) are selected to train the *NN*, whereas the excluded trial (Validation Set – *^j^VaS_i_*) is adopted to determine the optimal number of iterations. For each inner *LOOCV* iteration, the outputs of the *NN* box are the Bias (*^j^b_i_*) and the Weight matrices of the hidden (*^j^W_hi_*) and output (*^j^W_oi_*) layers, which are later used on the projected *TeS_i_* (*S′_i_*) to calculate the resultant vector *^j^out_i_*. Each time point of *^j^out_i_* is a dimensionless either *W*, *P*, or *N/A* (uncertainty) class. A potential perturbation is considered as detected if 5 consecutive time points are classified as *P*. A detected potential perturbation is then labelled as True Positive (*^j^TP_i_*), False Negative (*^j^FN_i_*), False Positive or True Negative (*^j^TN_i_*). Only in the *^j^TP_i_* case, the Detection Time (*^j^DT_i_*) is defined as the time elapsed from the delivery of the perturbation to the subject and the actual detection. Finally the Mean Detection Time (*MDT*) is defined as the average of all *^j^DT_i_* and the occurrences of *^j^TP_i_*, *^j^FP_i_*, *^j^TN_i_*, *^j^FN_i_* are counted (*TP_tot_*, *FP_tot_*, *TN_tot_*, *FN_tot_*). The *Sensitivity*, *Specificity* and *Accuracy* of the whole process are finally calculated as explained in *Materials and Methods*.

Figure 2 shows the overall algorithm used in this study.

A dataset *M* was constructed for each subject by concatenating data related to the 20 collected trials (i.e., 2 sessions×10 perturbations), each one ranging from five seconds before (Walking Phase: *WP*) to one second after (Perturbation Phase: *PP*) the onset of the perturbation, for a total of 600 data points (i.e., for each subject, *M* was sized as follows: [15 segments×3 acceleration components] rows; [20 trials×600 data points] columns). This dataset was preliminary parsed out by the *ICA* (see *ICA1* in Figure 2) in order to obtain the *Ranking* of the segments based on their informativeness and the optimal number *N* of retained Independent Components (*ICs*; see Appendix S1).

Then, the detection of the perturbation events was achieved by developing a machine learning algorithm consisting of two parts: extraction of the most informative components by the *ICA* (see *ICA2* in Figure 2) followed by pattern recognition implemented by a *NN*. The whole process was developed in compliance with leave-one-out cross validation (*LOOCV*) specifics.

#### Processing of the datasets accounting for all segments

For the dataset M, 19 out of 20 trials (Training Set: TrS_i_) were selected to train the model, whereas the excluded i^th^ trial (Test Set: TeS_i_) was adopted to test its performance. This procedure was repeated 20 times (“outer” LOOCV) leaving a different trial as TeS_i_ each time (i.e., 20 TrS_i_ and 20 TeS_i_ for each subject; see the green box in Figure 2).

The *ICA2* was performed on the *TrS_i_* with the *N* components previously determined. *ICA2* produced a time-invariant matrix (*W_ICAi_*, sized as follows: *N* rows; [15 segments×3 acceleration components] columns) which was used to project both the *TeS_i_* and the *TrS_i_* into the independent component space. The resultant projected *TrS_i_* (*S_i_*, sized as follows: *N* rows; [19 trials×600 data points] columns) was passed on to an artificial three-layer feedforward *NN* with 80 hidden neurons and tangent hyperbolic transfer functions (Neural Networks Toolbox, MATLAB®). The training function chosen was the “Resilient Backpropagation” algorithm (reported to be fast and appropriate when using sigmoid transfer functions) with “early stopping” feature to prevent overfitting issues (see Appendix S2) [28].

A further *LOOCV* step (inner *LOOCV*) was applied to *S_i_* (19 trials): 18 of them (*^j^S_i_*) were selected to train the *NN*, whereas the *j^th^* excluded trial (Validation Set – *^j^VaS_i_*) was used to determine the optimal number of iterations as the “early stopping” method suggests (see the red box in Figure 2).

For each inner *LOOCV* iteration, the outputs of the *NN* were both the Bias (*^j^b_i_*) and the Weight matrices of the hidden (*^j^W_hi_*) and the output (*^j^W_oi_*) layers, which were later used with an activity threshold of 0.5 on the projected *TeS_i_* (*S′_i_*) to calculate the resultant vector *^j^out_i_* (sized 1×600). Each individual time point of *^j^out_i_* was a dimensionless either *W* (Walking), *P* (Perturbation), or *N/A* (uncertainty) class.

A potential perturbation was considered as detected if five consecutive time points were classified as *P*. A detected potential perturbation was then labeled as “True Positive (*^j^TP_i_*)” if detected in the real *PP* or as “False Positive (*^j^FP_i_*)” if detected in the *WP*. Conversely a detected walking condition was labeled as “False Negative (*^j^FN_i_*)” if detected in the *PP* or as “True Negative (*^j^TN_i_*)” if detected in the real *WP*. Only in the *^j^TP_i_* case, the Detection Time (*^j^DT_i_*) was defined as the time elapsed from the delivery of the perturbation and the actual detection.

Finally, the Mean Detection Time (*MDT*) was defined as the average of all *^j^DT_i_* and the occurrences of *^j^TP_i_*, *^j^FP_i_*, *^j^TN_i_*, *^j^FN_i_* were counted (*TP_tot_*, *FP_tot_*, *TN_tot_*, *FN_tot_*). The *Sensitivity*, *Specificity* and *Accuracy* of the whole process were calculated as follows:
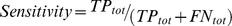


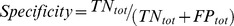


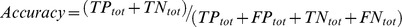
The algorithm run on the whole dataset *M* provided the reference performance.

#### Processing of datasets accounting for a subset of all body segments

The procedure described above was repeated accounting for all possible combinations of the most informative segments singled out by the Ranking performed by the initial ICA1 (see Figure 2). Specifically, the Total Segment Weight (TSW) was computed and each segment was ranked according to its informativeness (see Appendix S1). Moreover, since there is no way of knowing in advance which side is interested by the perturbation, segments were chosen bilaterally (i.e., belonging to both sides). The performance metrics (*MDT*, *Accuracy*, *Specificity*, and *Sensitivity*) were finally computed for each combination of selected body segments.

Noticeably, for this step, the number of retained *ICs* for each subject within the *ICA2* equalled the dataset dimension, that is the number of variables (number of segments×3 acceleration components). In fact, information reduction was already carried out by determining the meaningful segments.

### Statistical Analysis

The effect of direction (i.e., paired *NL/NR*, *NW/NE*, *W/E*, *SW/SE*, and *SL/SR*) and side (i.e., left and right foot) on the *MDT* obtained for each subject and each type of perturbation was analyzed using a two-way ANalysis Of VAriance (ANOVA). Finally the *MDT* obtained after processing datasets accounting for all body segments (*ALL*) was compared to those obtained with the reduced subsets by using the t-test on paired samples. Data analysis was carried out off-line by means of customized MATLAB (The MathWorks Inc., Cambridge, MA, US) scripts, and the statistical significance was set at *p*<0.05.

## Results

Subjects walked at an average speed of 1.10±0.03 m/s (range: 1.02–1.14 m/s). After the perturbation, all participants were able to recover their balance without falling.

**Figure 3:**
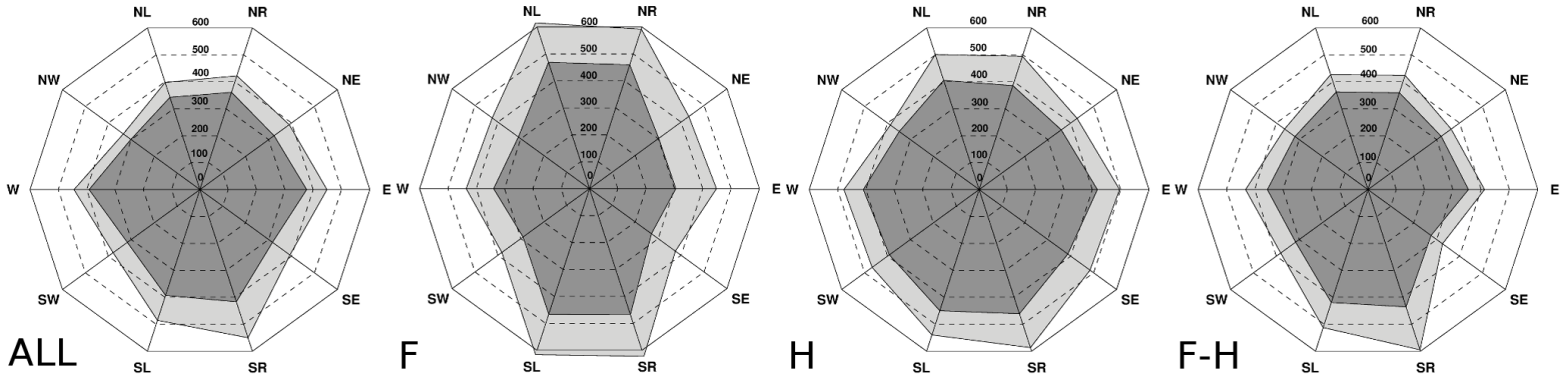
Mean Detection Time. The Figure shows the Mean Detection Time (*MDT*) obtained for each type of perturbation (i.e., *NR*, *NE*, *E*, *SE*, *SR*, *NL*, *NW*, *W*, *SW*, *SL*) averaged across all participants (dark gray area) plus one standard deviation (light gray area) considering the all-segments (*ALL*), the feet (*F*), the hands (*H*) and the feet-hands (*F-H*) combinations. All the values are expressed in *ms*.

Figure 3 shows the *MDT* for *ALL* considering each perturbation direction. On average, the *MDT* was 351±123 ms with an *Accuracy* of 95.4% (*Sensitivity* = 92.7% and *Specificity* = 98.0% – see Table 1).

**Table 1:** The table shows the Mean Detection Time (*MDT*), *Sensitivity*, *Specificity* and *Accuracy* for the all-segments combination (*ALL*) and the reduced-segments combinations chosen after the *Ranking*.

		*Factor:*	*Side*	*Factor:*	*Direction*				
	*MDT* [ms]	*p-value*	*F(1,149)*	*p-value*	*F(4,149)*	*t-test*	*Sensitivity* [%]	*Specificity* [%]	*Accuracy* [%]
*ALL*	351±123	0.417	0.662	**<0.001**	8.45	/	92.7	98.0	95.4
*F*	344±191	0.515	0.43	**<0.001**	14.49	0.347	84.8	98.0	91.4
*H*	399±128	0.939	0	**0.001**	4.97	**<0.001**	90.2	96.0	93.1
*F-H*	346±121	0.696	0.153	**<0.001**	11.60	0.789	92.1	96.3	94.2

The accounted combinations are feet (*F*), hands (*H*), and feet-hands (*F-H*). The p-values are related respectively to the two-ways ANOVA (i.e., effect of the direction and side of the perturbation) and the t-tests (i.e., difference of each reduced-segments combination with respect to the all-segments one) on the *MDT* obtained for each subject and each type of perturbation (see *Materials and Methods*). When a p-value is statistically significant (*p*<0.05), it is highlighted in bold.

The two-way ANOVA revealed that the *MDT* was significantly affected (*p*<0.05) by the perturbation direction but not by the side (Table 1). Noticeably, the perturbations that accounted for a diagonal movement of the belt (i.e. *NW*, *NE*, *SW* and *SE*) were the quickest to detect (Figure 3).

**Figure 4:**
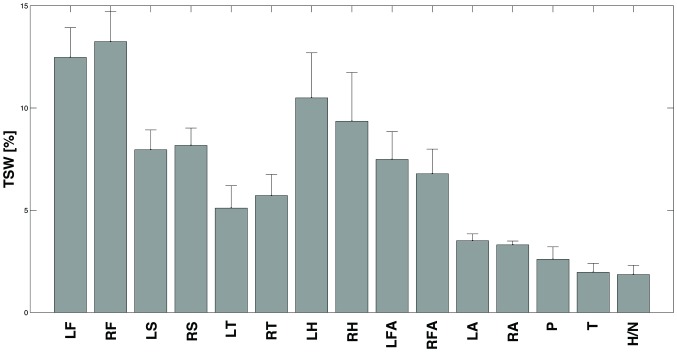
Total Segment Weight. The Figure shows the mean and one standard deviation (error bar) of the Total Segment Weight (*TSW*) of each body segment that is the cumulative weight of the accounted segment on the *ICs* extracted. The *TSW* was normalized and expressed as a percentage. The 15 segments are: head/neck (*H/N*), chest (*T*), abdomen/pelvis (*P*), upper arms (*LA* and *RA*), forearms (*LFA* and *RFA*), hands (*LH* and *RH*), thighs (*LT* and *RT*), shanks (*LS* and *RS*) and feet (*LF* and *RF*). See Appendix S1 for further details.

Figure 4 shows the *Ranking* performed by the *ICA1* process based on the informativeness of each body segment.

According to the *Ranking* information, the segments chosen for estimating the performance of the algorithm while processing data coming from a subset of body segments were feet (*LF*, *RF*) and hands (*LH*, *RH*). Consequently the accounted combinations were: feet (*F*), hands (*H*) and feet-hands (*F-H*). In this respect, the two-way ANOVA revealed again that the *MDT* was significantly affected (*p*<0.05) by the direction but not by the side of the perturbation (Table 1). Specifically, pure longitudinal (i.e., *NL*, *NR*, *SL* and *SR*) and transversal (i.e., *W* and *E*) perturbations were the longest to be detected respectively for the *F* and the *H* combinations (Figure 3). Accordingly, the concomitant contribution of both hands and feet (see *F-H* in Figure 3) to the algorithm induced a slight decrease of the *MDT* for all directions, involving results more similar to *ALL*. On the whole, the t-test showed that only the *MDT* of the *H* combination (399±128 ms) was significantly greater than that of *ALL* (*p*<0.05) (Table 1).

Concerning the statistical performance, when the algorithm processed data related to lower extremities (i.e., *F* combination), it worsened in *Sensitivity* (84.8%) but not in *Specificity* (98%), with an overall *Accuracy* of 91.4%. Furthermore, accounting for only hands (i.e., *H* combination) or feet and hands (i.e., *F-H* combination), the algorithm slightly worsened both in *Sensitivity* (respectively 90.2% and 92.1%) and *Specificity* (96% and 96.3%), with an overall *Accuracy* of 93.1% and 94.2%.

Concluding, the best-performance with a reduced-segment combination was obtained by processing data related to the *F-H*, subset and resulted in an *Accuracy* of 94.2% and a *MDT* of 346±121 ms.

## Discussion

In this study we investigated how to optimize sensor positioning in order to improve the performance of pre-impact fall detectors while discriminating walking from falling processes. Specifically, we hypothesized that the modifications of the linear acceleration of a subset of body segments can be more sensitive than others in successfully and quickly sorting walking from unexpected multi-directional slipping-like events. The performance of our ad-hoc-designed machine learning algorithm was tested on different subsets taken from the available data with a view to determining both the best obtainable performance and the minimum number of segments needed to maintain good classification performance.

Results showed that the performances obtained when taking into account the greatest redundancy among data (i.e., when observing all body segments–*ALL* combination) were: *MDT* = 351±123 ms and *Accuracy* = 95.4% (Table 1). Moreover, feet and hands were the body segments accounting for the greatest amount of informativeness (Figure 4) and, as expected, the detection of the perturbation based on the combination of them was only slightly worsened (Table 1).

Our analysis suggests that although whole-body reaction is important when both walking [29] and managing unexpected perturbations [30], [31], the kinematics of feet and hands during sudden postural transitions is characterized by the widest variation. It is presumably due to their lower inertia and higher distance from the body *CoM*, and documents that these body segments are very sensitive to the modifications of the dynamic stability induced by slipping-like perturbations.

Results were consistent with findings of previous authors [22], who observed that an *a posteriori* classifier based on the 3D acceleration of three markers (the left ankle, right ankle and sternum) performed better in distinguishing falls due to slips, trips and other causes than using a higher number of them. This confirms that the information conveyed by all the segments is redundant and does not increase classification performance.

Interestingly, the absence of data related to the feet worsened the performance of the algorithm, increasing significantly (p<0.05) the *MDT* (Table 1), in agreement with Zhang and colleagues [13] who found the acceleration of the prosthetic foot to be sufficient to detect stumbles with a fast-time response compared to others variables (e.g., EMG signals, knee angle acceleration, ground reaction force, *CoM*-*CoP* inclination angle etc.). Feet are indeed the first segments to undergo modifications of the steady biomechanical patterns due to slipping-like perturbations. In this respect, although upper limb reactions occur at similar latencies as lower limbs reactions [31], the dynamics of the mechanical chain linking the lower and the upper limbs may introduce a delay which negatively affects the performance of the fall detection algorithm.

It is worth noting that the statistical performance of the algorithm while processing only data related to the feet (*F* configuration) resulted in a reduced *Sensitivity* due to a higher number of *FN_tot_*. The contribution of the hands can hence be helpful to reduce false alarms. Overall, the optimal trade-off between good statistical performance and low *MDT* can be obtained by processing data related to both the upper and lower distal extremities.

As expected, results also showed that the *MDT* for all accounted combinations of body segments was significantly affected by the direction (i.e., paired *NL/NR*, *NW/NE*, *W/E*, *SW/SE*, and *SL/SR*) but not by the side (i.e., left or right foot) of the perturbation. This confirms the need to analyze reactions to unexpected slipping perturbations in several directions given the context-dependent reactive responses elicited by subjects and the unpredictability of real life fall direction [19], [23].

If we analyze the state of the art for pre-impact fall detection, previous authors have reported higher values of *Sensitivity* and/or *Specificity* [6], [7], [9], [12]. Actually, we believe that our results cannot be directly compared to those reported in literature for the following three main reasons. First, it is common practice to impose a free fall on standing-still subjects [6], [7], [8], [9], [11], [12] that results in an abrupt change of the dynamical features. Instead, the protocol adopted in this work consisted in delivering an unexpected slipping-like perturbation during steady walking inducing less blatant modifications of the biomechanical patterns. Second, the double *LOOCV* used to validate the algorithm rendered the results more conservative and better generalizable compared to other approaches [32]. This was because the *TeS* of each *LOOCV* cycle was always unknown to the algorithm thus making the results more pessimistic but closer to real-life performance. Finally, in previous papers, authors have analyzed either the “lead time” (i.e., the time elapsed from the fall detection and the actual impact of the subject on the mattress) [6], [7], [8], [9], [10], [11], [12], or the critical timing of falling (i.e., the time elapsed from the detection of a fall and the moment at which the *CoM*-*CoP* inclination angle exceeded a range of −23° to 23° from vertical) [13]. On the contrary, in this study, we analyzed the time elapsed from the onset of the perturbation and the actual detection, that is, our attention was focused on the kinematics of the body segments in the time window preceding that observed by previous authors.

Concerning the methodological aspects, while current pre-fall detection approaches compare daily-activity kinematic features to those related to simulated falls with threshold-based algorithms [6], [7], [8], [9], [10], [11], [12], [13], [21], for the first time a machine learning approach was used to identify the combination of body segments which provides for a rapid and reliable detection of the transition from steady walking to a slipping event. Specifically, the information was firstly disentangled from noise using a reliable *ICA* algorithm on linear accelerations, then the *NN* classifier was fed with correctly-preprocessed and informative data.

Indeed, we have to acknowledge that there are many possible techniques for dimensionality reduction (e.g., *ICA*, Principal Component Analysis, Nonnegative Matrix Factorization, Factor Analysis, etc.) and classification (e.g., *NN*, Linear Discriminant Analysis, Support Vector Machines, Hidden Markov Models, etc.). However, determining the combination of techniques that provide the absolute best results is out of the scope of this work.

We would like to remark that our approach, at this stage, was not designed or suited to work in real time but was rather aimed to determine the optimal positioning of a limited number of sensors. Nonetheless, its output can be potentially applied in a real-time pre-impact fall detector. For instance, the model could be firstly trained off-line based on data recorded during daily activities and later used on-line to identify the current state of the subject. Under this hypothesis, since the time interval between the loss of balance during the quite upright stance and the impact with the ground is longer than about 0.7 s [22], [33], it is possible to speculate that our approach may allow early enabling of several fall prevention strategies.

Finally, it is important to pinpoint that linear accelerations were not recorded by inertial units but were estimated from the 3D kinematics of the whole body by using a well standardized motion analysis approach, which is not applicable in unconstrained environments. Nonetheless, the outcome of this study can possibly be extended to an inertial unit-based approach due to the algebraic relationship between accelerations estimated with respect to a fixed and a mobile reference frame. Accordingly, the presented results are expected to be well suited for all widely used real-time fall detection systems based on inertial units.

### Limits of the study

As all the experimental approaches used in literature, the one proposed in this work is not fully generalizable. In fact, even if the adopted paradigm is more realistic compared to other works [6], [7], [8], [9], it is still constrained and real life falls may differ from artificially-induced ones. Moreover we investigated the behaviour of our algorithm while processing only one type of daily living activity (i.e., walking) and one type of falling cause (i.e., slipping).

Another limitation is that all the experiments were performed by healthy young subjects. It is well know that elderly people (i.e., natural users of pre-impact fall detectors) have a falling dynamics different to young subjects due to their residual physical/cognitive capabilities [34], [35]. Therefore, further experimental sessions are required to verify whether the results obtained in this study can be extended to older subjects. However the analysis of the reaction of healthy young subjects is the first and necessary step to identify an approach on which to base further studies on older persons and to proceed further to the goal of providing effective fall protection devices.

On the whole, further experiments aimed at monitoring several daily activities (e.g., sit-to-stand, stair ascending and descending, laying, etc.) and other falling causes (e.g., trips, miss-steps) in both young and elderly people are required to assess the extent to which such results can be reproduced in older adults and in different experimental conditions.

## Conclusions

The paper shows how to optimize sensory positioning with a view to improving the performance of pre-impact fall detectors. Results confirm the hypothesis that the linear acceleration of only a limited set of body segments can be very sensitive to underlying fall-triggering processes in healthy young adults. On the whole, in the framework of the proposed approach, the information conveyed by all the body segments is redundant to achieve effective fall detection, and suitable performance, in terms of *MDT* and *Accuracy*, can be obtained only by observing feet and hands. These results could serve as a guideline to improve the performance of pre-impact fall detectors based on an optimized positioning of the sensors.

## Supporting Information

Appendix S1 (DOC)

Appendix S2 (DOC)
