# Supplementary material for: Pre-Impact Fall Detection: Optimal Sensor Positioning Based on a Machine Learning Paradigm
Source: PLoS One. 2014 Mar 21;9(3):e92037. doi: 10.1371/journal.pone.0092037 (PMC3962372; doi:10.1371/journal.pone.0092037)
Supplement: Appendix S1 — (DOC) [file pone.0092037.s001.doc]

## Appendix S1: Independent Component Analysis and segment reduction

The *ICA* technique was used to parse multivariate data into maximally-independent components by a linear transformation in order to feed the classifier and improve its performance. Specifically, *ICA* maximized independence using all the information brought by the probability density distribution of the input data and disentangling information from noise . Briefly, *ICA* computes a *m x n* full rank “unmixing matrix” *W* that linearly projects a *n*-variables dataset (i.e., the acceleration of all body segments), *x(t)*, onto a *m*-dimensional one, *s(t)*, such that *s(t) = W x(t)*. *W* is a weight matrix that describes the contribution of the *m* *ICs* on each of the *n* variables. To select the most reliable and informative *ICs* in this work an improved version of the *FASTICA* algorithm , which combines trial-to-trial bootstrapping and algorithm-starting-point randomization to increase *ICs* reliability , was used.

The adopted *ICA* algorithm was also able to provide both the optimal number of *ICs* to retain (*N*) and the *Ranking* of the body segments based on their informativeness. Specifically, the *ICA* was run 150 times from 150 randomized starting points, and the trials were bootstrapped with replacement. The optimal number of *ICs* to retain (*N*) was chosen according to the quality index *Iq* that measured the repeatability of each component across the 150 runs: in order to pass only meaningful data to the classifier, the noisier components (i.e., more subjected to variability across runs–with a low *Iq*) were excluded while maintaining a sufficient amount of variance explained (above 85%).

The *Ranking* of the body segments was achieved as follows. The weight that each input variable had on each extracted *IC* was analyzed and its absolute value (Variable Weight - *VW*) was computed. Then, the Segment Weight (*SW*) was calculated as the sum of the *VWs* corresponding respectively to the *X*, *Y*, and *Z* acceleration components of the segment. The cumulative segment weight (*CSW*) was defined as the sum of the *SWs* for all the retained *ICs*. To account for differences in explained variance across subjects, the values of *A* (pseudoinverse of *W*) were normalized so that the sum of all the *CSWs* equaled 100% (divided by the sum of *CSWs* and multiplied by 100). The *CSW* of the *j^th^* segment can be also seen as the sum of the l_1_ norms (which has the advantage of limiting outliers influence) of the rows of *A* corresponding to the *j^th^* segment. Finally the Total Segment Weight (*TSW*) of each segment was estimated as the average of the *CSW* across all the subjects. In conclusion, the *TSW* represents the cumulative weight of the accounted segment on the *ICs* extracted, i.e., its *Ranking*.

On the whole, the adopted *ICA* algorithm was able to perform three tasks: (i) to extract the optimal number of *ICs* to retain (*N*); (ii) to rank the input data based on their informativeness; (iii) to return the unmixing matrix *W*. Noticeably, it is possible to by-pass the task (i) by giving the number of *ICs* to retain as input. Since the task (i) is very computationally intensive, in the framework of this study, the *ICA* was performed in two steps (Figure 2): *ICA1* was run once on the whole dataset M accounting for 20 trials and achieved tasks (i) and (ii); ICA2 was run within the outer *LOOCV* and achieved the task (iii) after giving the number of *ICs* to retain as input in accordance with the dataset being processed. Specifically:

- the dataset accounting for all body segments was parsed out by *ICA2* where the number of retained *ICs* was *N*;
- the dataset accounting for a subset of all the body segments, identified by the *Ranking*, was parsed out by *ICA2* where the number of retained *ICs* equalled the dataset dimension.

# References

1. Hyvarinen A (1999) Fast and robust fixed-point algorithms for independent component analysis. IEEE Trans Neural Netw 10: 626-634.

2. Artoni F, A. Gemignani, L. Sebastiani, R. Bedini, A. Landi, D. Menicucci. ErpICASSO: a Tool for Reliability Estimates of Independent Components in EEG Event-Related Analysis; 2012; San Diego, CA, USA.

3. Himberg J, Hyvarinen A, Esposito F (2004) Validating the independent components of neuroimaging time series via clustering and visualization. Neuroimage 22: 1214-1222.
