# Supplementary material for: Pre-Impact Fall Detection: Optimal Sensor Positioning Based on a Machine Learning Paradigm
Source: PLoS One. 2014 Mar 21;9(3):e92037. doi: 10.1371/journal.pone.0092037 (PMC3962372; doi:10.1371/journal.pone.0092037)
Supplement: Appendix S2 — (DOC) [file pone.0092037.s002.doc]

## Appendix S2: Neural Networks

In this work, a multilayer feedforward *NN* trained in batch mode with one input layer, one hidden layer and one output layer was used. Tangent hyperbolic transfer functions were used both in the hidden and output layers. The “Resilient Backpropagation algorithm” was chosen as training function with “early stopping” feature: the training stopped at the minimum error on the validation set (*^j^VaS_i_* in Figure 2), that is, whenever the gradient of performance did not increase for 6 iterations consecutively. The number of hidden layer neurons (i.e., 80) and the number of *P* time points needed to detect a potential perturbation (i.e., 5) were determined in a preliminary data analysis varying the number of hidden nodes from 20 to 90 and the time window from 1 to 10 while minimizing the overall misclassification on the validation sets. The initial values of weights and biases were given by the Nguyen-Widrow method [28]. The mean square error, that is the average square error between the network outputs and the target outputs, was chosen as performance function. Two output neurons were used, one representing walking (*W*) and one representing perturbations (*P*). The node with the highest output signal was labelled as winning neuron (i.e., the class to be assigned to *^j^out_i_*). If neither of the output neurons exceed the activity threshold (set at 0.5) an uncertainty class (*N/A*) was assigned.
